# Preparation of a Novel Fe/Ca Modified Chlorella Biochar for Phosphorus Removal from Mariculture Tail Water by Response Surface Methodology

**DOI:** 10.3390/ma19091700

**Published:** 2026-04-23

**Authors:** Kehan Yu, Haifeng Jiao, Changjun Liu, Dan Zheng, Xiafei Zheng, Yurong Zhang, Xizhi Shi

**Affiliations:** 1 School of Biology and Environment, Zhejiang Wanli University, Ningbo 315100, China; 19883006743@163.com (K.Y.); zhengxiafei@hotmail.com (X.Z.); zhangyurong@zwu.edu.cn (Y.Z.); 2 Xiangshan Aquatic Technology Promotion Station, Ningbo 315731, China; xs65763110@126.com; 3 Ningbo Institute of Ocean and Fisheries, Ningbo 315048, China; newstart_88@sina.com; 4 School of Marine Sciences, Ningbo University, Ningbo 315211, China; shixizhi@nbu.edu.cn

**Keywords:** mariculture tail water, iron–calcium modification, chlorella biochar, phosphorus removal, response surface optimization, adsorption mechanism

## Abstract

Excessive phosphorus discharge from aquaculture effluent significantly contributes to coastal eutrophication, while conventional adsorbents exhibit limited phosphorus removal efficiency in high-salinity, weakly alkaline seawater effluent. This study developed iron/calcium co-modified chlorella biochar (FCBC) through co-impregnation and high-temperature pyrolysis, optimizing the preparation process via the Box–Behnken response surface method. The optimal conditions were identified as an iron concentration of 2.5 mol/L, a calcium concentration of 2.0 mol/L, a pyrolysis temperature of 717 °C, and a duration of 113 min. Under these conditions, FCBC achieved a phosphorus removal rate of 93.23% within 3 h, which was significantly higher than that of the unmodified Chlorella biochar (BC, <8% within the same reaction time). The Fe/Ca co-modification endowed FCBC with a positively charged surface, an increased average pore size of 22.773 nm, and good magnetic responsiveness (saturation magnetization of 6.68 emu·g^−1^). FCBC demonstrated remarkable adaptability, achieving over 97% phosphorus removal across a pH range of 3 to 11, salinity levels of 5 to 40‰, and phosphorus concentrations of 1 to 15 mg/L. Its adsorption kinetics conformed to pseudo-second-order kinetics (R^2^ = 0.987) and the Freundlich model (R^2^ = 0.971), with efficient phosphorus removal primarily attributed to iron–calcium synergistic effects. FCBC presents significant potential for phosphorus treatment in marine aquaculture effluents.

## 1. Introduction

Marine aquaculture is a critical contributor to the global seafood supply and economic development [1,2]. However, intensive farming practices result in approximately 20–30% of feed phosphorus remaining unassimilated by cultured organisms, ultimately discharging into coastal waters as dissolved or particulate pollutants [3]. Elevated phosphate inputs stimulate algal blooms, disrupt ecosystem balance, and exacerbate eutrophication, manifesting as frequent red tides, dissolved oxygen depletion, and ecological degradation of nearshore habitats [4,5,6]. Consequently, efficient phosphorus removal from aquaculture effluent is imperative for advancing sustainable marine aquaculture.

Among available treatment technologies, adsorption is widely regarded as a promising approach due to its operational simplicity, cost-effectiveness, and environmental compatibility [7]. Biochar, particularly when derived from microalgae, has garnered considerable interest as an adsorbent owing to its porous architecture, high specific surface area, and chemically tunable surface [8,9,10,11,12,13,14]. Nonetheless, pristine biochar typically possesses a negatively charged surface, which restricts its affinity toward anionic phosphate species [15]. Iron modification has been shown to enhance positive charge density and anion exchange capacity [16,17,18]; however, iron-modified biochar suffers from competitive anion interference (e.g., Cl^−^, SO_4_^2−^) and active component leaching in high-salinity seawater, substantially compromising phosphorus removal efficiency [19,20]. Conversely, calcium-modified biochar offers improved stability in saline environments but exhibits sluggish adsorption kinetics and is susceptible to OH^−^ competition under alkaline conditions [21,22,23,24].

Recent advances suggest that iron–calcium composite modification can synergistically address these limitations by integrating the rapid adsorption kinetics of iron with the chemical robustness of calcium, thereby achieving broad pH tolerance and sustained phosphorus uptake in complex aqueous matrices [25,26]. For instance, Wang et al. [27] demonstrated that Ca/Fe-modified biochar synthesized at 700 °C possessed a highly ordered porous structure and achieved a maximum phosphate adsorption capacity of 890.8 mg·g^−1^, with adsorption behavior conforming to pseudo-second-order kinetics and the Sips isotherm model. A comprehensive review by Gao et al. [28] further underscored the emerging paradigm of employing metal-synergized composites for enhanced phosphorus sequestration. Additionally, an innovative study demonstrated that concentrated seawater could serve as a source of Ca^2+^ and Mg^2+^ for biochar modification [29]. This research indicated that seawater-derived metal ions could be effectively incorporated into biochar matrices, resulting in a phosphate adsorption capacity of 138.2 mg·g^−1^, thereby suggesting the potential applicability of such materials in marine environments. Despite these advances, the systematic optimization of iron–calcium co-modified microalgae biochar specifically tailored for phosphorus removal from mariculture tail water—a high-salinity, weakly alkaline, and chemically intricate medium—remains unexplored.

In this study, Chlorella was selected as the biochar precursor, and the preparation conditions were optimized using Box–Behnken response surface methodology. Key parameters, including Fe/Ca concentration, pyrolysis temperature, and pyrolysis time, were systematically investigated to develop a novel Fe/Ca modified Chlorella biochar (FCBC). The phosphorus removal performance of FCBC was evaluated using simulated mariculture tail water, and the underlying adsorption mechanisms were elucidated through comprehensive physicochemical characterization (SEM-EDS, BET, XRD, XPS, FTIR, VSM, zeta potential) coupled with kinetic and isotherm modeling. This work aims to provide a cost-effective, high-performance adsorbent tailored for phosphorus remediation in marine aquaculture effluent, thereby facilitating the valorization of microalgal waste and contributing to the sustainable development of coastal aquaculture.

## 2. Materials and Methods

### 2.1. Materials

Chlorella was thoroughly washed, freeze-dried, ground into fine powder, and then passed through a 100 mesh sieve.

The following substances used in the study: anhydrous ferric chloride (FeCl_3_, ≥99%), anhydrous calcium chloride (CaCl_2_, 96%), polyethylene glycol 4000 (PEG 4000, ≥99%), Flaky sodium hydroxide (NaOH, ≥98%), hydrochloric acid (HCl, 20%), ammonium molybdate solution ((NH_4_)_6_Mo_7_O_24_∙4H_2_O, 1.3 g/L), sulfuric acid (H_2_SO_4_, 1.84 g/mL), antimony potassium tartrate solution (C_4_H_4_KO_7_Sb∙0.5H_2_O, 35 mg/L), L-ascorbic acid solution (10%), sulfonamide (C_6_H_8_N_2_O_2_S, ≥99%), potassium dihydrogen phosphate solution (KH_2_PO_4_, 2 µg/mL), and chloroform (CHCl_3_, ≥99%).

The purity of the above reagents was analytical grade and purchased from Sinopharm group and Shanghai McLean Biochemical Technology Co., Ltd. (Shanghai, China). Deionized water was used as experimental solvent.

### 2.2. Synthesis of FCBC

The Chlorella pyrenoidosa algal strain underwent a series of purification procedures, which included concentration, washing, filtration, and impurity removal, ultimately leading to freeze-drying. The resulting Chlorella biomass powder was ground in a grinder and then sieved through a 100-mesh screen for further application. The Chlorella biomass powder was subsequently modified using the impregnation method, followed by pyrolysis to yield FCBC. To improve the loading capacity for iron and calcium, EDTA was chosen as the chelating agent during the preparation process.

The preparation process for the FCBC is detailed as follows: The mixture comprises Chlorella biomass powder, 0.04 g of polyethylene glycol (PEG 4000), and solutions of iron chloride and calcium chloride at specified concentrations. At ambient temperature, the mixture was continuously stirred with a magnetic stirrer at 300 r/min until it achieved a uniform paste consistency. Subsequently, the mixture was covered with plastic wrap and allowed to soak for a minimum of 48 h. After thorough mixing, the solution was transferred to a 50 mL centrifuge tube and subjected to centrifugation at 4000 revolutions per minute for 15 min. Following centrifugation, the upper solid layer was extracted and dried in an oven at 80 °C for 12 h. The dried solid was then placed in a quartz boat and introduced into a tube furnace. Nitrogen gas was introduced into the tube at a flow rate of 20 mL/min for 10 min to establish an anaerobic environment. The pyrolysis temperature was increased to the set point at a controlled heating rate of 10 °C/min, maintained for a specified duration, and subsequently cooled at 10 °C/min to 400 °C. After a natural cooling period to room temperature, the modified biochar was retrieved and designated as FCBC.

Concurrently, pure Chlorella biochar (BC) was prepared as a control under identical conditions as FCBC.

### 2.3. Response Surface Methodology for Optimizing FCBC Preparation

Response surface methodology (RSM) serves as an effective statistical tool for optimizing multivariate processes while minimizing the number of experiments required. In this study, the Box–Behnken design (BBD) was selected over the central composite design (CCD) due to its requirement for fewer experimental runs (30 runs for BBD compared to 30–50 runs for CCD) and its capacity to avoid extreme factor combinations that could yield suboptimal biochar [27]. Furthermore, RSM offers superior interpretability of factor interactions compared to artificial intelligence models, such as neural networks, making it more suitable for typical small datasets encountered in laboratory optimization [28].

In previous experiments, modified materials fired at 300 °C to 900 °C have been made and phosphorus removal effects have been compared. The factor level of the response surface model was selected according to preliminary experiments and previous studies [30]. The present study utilized the BBD method to assess the phosphorus removal rate (%, Y) from artificially simulated wastewater using FCBC as the response variable. This approach aimed to investigate the interactions among variables and to identify the optimal parameters for FCBC preparation through Response Surface Methodology (RSM). Four parameters were selected for the experimental design: the concentration of the Fe/Ca impregnation solution, pyrolysis temperature, and pyrolysis time. Each parameter was assigned three levels: high (+1), central (0), and low (−1). These parameters facilitated the optimization coding and variation range of the response surface method, as detailed in Table 1. Each experimental condition was replicated three times, and the response values were reported as the mean. The number of experiments is dictated by the Box–Behnken design (BBD), which adheres to the principles of statistical efficacy, practical feasibility, and the overarching guidelines of response surface methodology (RSM) optimization. BBD is classified as a second-order design that incorporates either rotation or approximate rotation. For k factors, the requisite number of experiments is calculated as 2k(k − 1) plus repetitions at the center point. In this study, with k set to 4, the number of basic experiments amounts to 2 × 4 × (4 − 1) = 24, in addition to 5 central points, resulting in a total of 29 experiments. However, in practice, the design is typically adjusted to 30 experiments (including the 5 center points) to enhance model stability [31].

The experimental data were fitted to a quadratic polynomial model using Design-Expert 13.0. Analysis of variance (ANOVA) was performed to evaluate the significance of each term and the model’s adequacy. The optimal conditions were determined by solving the regression equation, and the predicted response was verified experimentally.

### 2.4. Adsorption Experiment of FCBC on Phosphorus

All adsorption experiments were conducted using simulated mariculture tail water prepared by dissolving sea salt crystals and KH_2_PO_4_ in deionized water. The pH was adjusted to 7.5–8.0 (typical of mariculture tail water) using NaOH and HCl. A fixed amount of FCBC was added to 100 mL of phosphate solution in 250 mL conical flasks. The mixtures were shaken at 180 rpm and 25 °C for a specified time. After adsorption, samples were filtered through qualitative filter paper (7–8 µm pore size), and the residual phosphate concentration was determined by the ammonium molybdate spectrophotometric method.

#### 2.4.1. Dosage

Weigh FCBC samples of varying masses (0.02, 0.04, 0.06, 0.08, 0.1, 0.15, 0.2 g) and transfer them into conical flasks. Prepare artificial simulated high-phosphorus seawater aquaculture tail water by dissolving sea salt crystals and potassium dihydrogen phosphate. Adjust the pH of the resulting solution to 7.5–8, which corresponds to the pH typical of conventional seawater aquaculture tail water, using NaOH and HCl solutions.

#### 2.4.2. Initial Phosphorus Concentration

Prepare conical flasks containing PO_4_^3−^-P solutions at varying mass concentrations (1, 2.5, 5, 10, 15, 20, 25 mg/L). Adjust the pH of each solution to 7.5–8 using NaOH and HCl solutions, and then add 0.1 g of FCBC to each flask.

#### 2.4.3. pH

Artificially simulated high-phosphorus mariculture tail water was prepared using sea salt and potassium dihydrogen phosphate, with pH levels adjusted to 3, 5, 7, 8, 9, and 11 using NaOH and HCl solutions. Equal volumes were transferred into conical flasks, to which 0.1 g of FCBC was added in each case.

#### 2.4.4. Salinity

Artificially simulated mariculture tail water with varying salinities (5, 10, 15, 20, 25, 30, 35, 40‰) was prepared using sea salt, and the total phosphorus concentration was subsequently measured. The simulated tail water was then adjusted to a uniform phosphorus concentration using potassium dihydrogen phosphate, and equal volumes were transferred into conical flasks. The pH of each solution was adjusted to 7.5–8.0 using NaOH and HCl solutions, followed by the addition of 0.1 g of FCBC to each flask.

#### 2.4.5. Adsorption Kinetics

The biochar dosage was set at 1 g/L, with an initial mass concentration of PO_4_^3−^-P at 50 mg/L. The pH was adjusted to a range of 7.5 to 8, and the temperature was maintained at 25 °C. The reaction occurred on a shaking table at 180 RPM for 3 h. Samples were collected at intervals of 5, 10, 20, 40, 60, 90, 120, 240, 560, 690, 1440, 1800, 2880, and 4320 min to assess the phosphorus concentration.

#### 2.4.6. Adsorption Isotherm

Prepare PO_4_^3−^-P solutions at concentrations of 5, 10, 20, 50, 100, 200, 500, and 1000 mg/L, adjusting the pH to a range of 7.5 to 8. Transfer 100 mL of each phosphate solution with varying mass concentrations into a 250 mL conical flask. Weigh 0.1 g of FCBC and add it to the conical flask. Allow the mixture to react at 25 °C for 24 h on a shaking table set at 180 RPM to determine the phosphorus concentration.

### 2.5. Analytical Method

#### 2.5.1. Calculation of Equilibrium Adsorption Capacity and Removal Rate

The phosphorus concentration is measured by the UV spectrophotometer. The phosphorus content (*q_e_*) and phosphorus removal rate (*η*) adsorbed on the biochar under the adsorption equilibrium state are calculated based on Equations (1) and (2) respectively, where *q_e_* (mg/g) represents the equilibrium adsorption capacity. η (%) is the phosphorus removal rate of biochar. C_0_ and C_e_ (mg/L) represent the mass concentration of PO_4_^3−^-P at the initial and equilibrium adsorption stages, respectively. V (L) is the solution volume. *m* (g) is the mass of biochar added.

(1) $$q_{e}=(C_{0}\;-\;C_{e})\;\times \;V/m$$

(2) $$\eta =(C_{0}{-C}_{e})/C_{0}\;\times \;100\%$$

#### 2.5.2. Adsorption Kinetics Models

The following three kinetic models were used for fitting. These include pseudo-first-order kinetics, pseudo-second-order kinetics, and Elovich kinetic model. The results were calculated based on Equations (3)–(5),where $q_{t}$ (mg/g) is the adsorption capacity of the adsorbent at time *t*. *t* (h) is the reaction time. *k*_1_ (h^−1^) and *k*_2_ (g/mg∙h) are the rate constants of pseudo-first-order kinetic model and pseudo-second-order kinetic model. α (mg/g∙min) is the initial adsorption rate. β (g/mg) is the chemisorption energy.

Pseudo-first-order kinetic model:

(3) $$\ln(q_{e}\;-\;q_{t})\;=\;\ln q_{e}\;-\;k_{1}t$$

Pseudo-second-order kinetic model:

(4) $$t/q_{t}\;=\;t/q_{e}\;+\;1/k_{2}{q_{e}}^{2}$$

Elovich kinetic model:

(5) $$q_{t}\;=\;(\ln\alpha \beta )/\beta \;\;+\;(\ln t)/\beta$$

#### 2.5.3. Adsorption Isotherm Models

Langmuir and Freundlich isotherm models were used to fit the adsorption process.

Langmuir model generally assumes that the adsorption is monolayer adsorption, and the result is calculated by Equation (6),where $q_{m}$ (mg/g) is the maximum adsorption capacity. *K_L_* (L/g) is the Langmuir adsorption constant.

(6) $$q_{e}=K_{L}C_{e}q_{m}/(1+K_{L}C_{e})$$

Freundlich model generally assumes that the adsorption is multi-layer adsorption, and the target pollutant is adsorbed on the heterogeneous adsorbent surface. The result is calculated by Equation (7),where *K_F_* (L^1/*n*^∙mg^1−1/*n*^/g) is Freundlich adsorption constant. 1/*n* is the adsorption strength.

(7) $$\ln q_{e}=\ln K_{F}+(\ln C_{e})/n$$

Langmuir and Freundlich models were selected because they are the most commonly used isotherm models in phosphate adsorption studies, providing insight into surface homogeneity/heterogeneity and adsorption capacity [32].

### 2.6. Material Characterization

The specific method of material characterization is shown in Table 2.

## 3. Results

### 3.1. Optimization of FCBC Preparation by Response Surface Methodology

#### 3.1.1. Model Fitting and ANOVA

A total of 30 operational groups, including five replicates of the center point, were designed. The results from the Box–Behnken design are presented in Table A1. These results were regressed and fitted using Design Expert 13. The objective function was the phosphorus removal rate (%, Y), leading to the derivation of the quadratic multiple regression Equation (8). In this context, A, B, C, and D represent the concentrations of Fe (mol·L^−1^), Ca (mol·L^−1^), pyrolysis temperature (°C), and pyrolysis time (min), respectively, along with the corresponding phosphate removal rate (%, Y).

(8) $$\begin{array}{c} Y=88.72+7.66A+9.45B+7.08C\;-\;1.17D\;-\;8.33\mathrm{AB}\;-\;1.15\mathrm{AC}\;-\;0.2927\mathrm{AD}\;-\;3.57\mathrm{BC}\\ -0.2148\mathrm{BD}+0.8706\mathrm{CD}\;-{\;6.18A\;}^{2}\;-\;{4.39B\;}^{2\;}-{\;4.10C\;}^{2\;}-\;{1.42D\;}^{2} \end{array}$$

ANOVA was conducted on the regression equation to validate the correlation and adequacy of the quadratic model. The findings from the ANOVA and the significance tests of the regression coefficients are presented in Table 3.

The F value indicates the significance level associated with the alignment between the model and the experimental data. A higher F value signifies a more substantial effect on the response variable and an improved fit [31]. The analysis revealed that the model’s F value was 57.13, with a calculated *p* value of <0.0001, confirming the statistical significance of the surface model. Among the factors analyzed, A, B, C, AB, A^2^, B^2^, and C^2^ were highly significant (*p* < 0.0001), while BC and D^2^ were significant (*p* < 0.05). Other factors did not reach significance (*p* > 0.05). The mismatch *p* value of 0.0987 indicates that the regression model is significant and closely aligns with the actual data, making it suitable for predicting response variables. The results of the model simulation analysis are summarized in Appendix A (Table A2), which reports an adjusted determination coefficient R^2^ of 0.9644 and a prediction coefficient R^2^ of 0.9044. The model’s accuracy, as indicated by the coefficient of variation (C.V.), is 4.10%, which is well below the 10% threshold, suggesting high accuracy. Furthermore, the signal-to-noise ratio (SNR) was 25.5403, significantly exceeding the threshold of 4, indicating good variance fitting and reliability.

The F value for each factor in the regression model indicates its relative importance in influencing the phosphorus removal rate. Based on the comparison of F values, it can be concluded that the concentration of Ca in the impregnation solution exerts the most substantial effect on the phosphorus adsorption process. Consequently, the influence of various factors on the phosphorus adsorption performance of FCBC follows the order: Ca concentration > Fe concentration > pyrolysis temperature > pyrolysis time. Regarding interactions, the hierarchy is as follows: AB > BC > AC > CD > AD > BD.

To further validate the adequacy of the quadratic model, comprehensive residual diagnostics were conducted.

**Figure A1:**
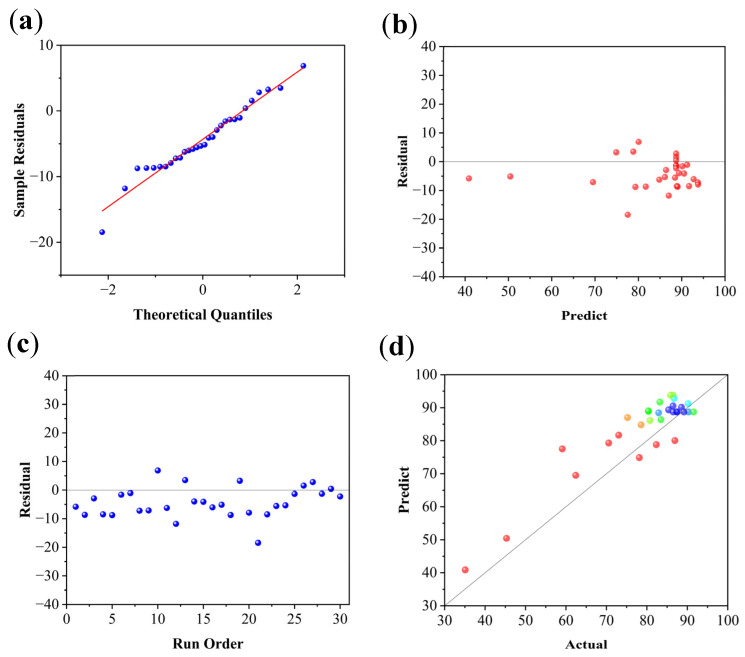
Residual diagnostic plots for the RSM model: (**a**) normal probability plot of residuals; (**b**) residuals vs. predicted values; (**c**) residuals vs. run order; (**d**) actual vs. predicted values with Y = X reference line. In (**a**), the line segment is the fitted line; in (**b**,**c**), the horizontal lines are the reference lines at Y = 0; in (**d**), points of the same color denote groups with similar synthesis conditions.

The residuals, defined as the differences between experimental and predicted values, were assessed through four diagnostic plots presented in Appendix A (Figure A1). The normal probability plot of residuals (Figure A1a) closely approximates a straight line, with a fitted linear equation of Residual = −4.322 + 5.126 × Theoretical Quantile (R^2^ = 0.964). This observation indicates that the residuals are normally distributed, thereby satisfying the normality assumption for ANOVA. The plot of residuals versus predicted values (Figure A1b) reveals a random scatter around zero, devoid of any evident funnel-shaped or curved pattern, which confirms constant variance (homoscedasticity) and the absence of systematic model errors. The residuals versus run order plot (Figure A1c) displays no significant trend, suggesting that the experiments were conducted under stable conditions without time-dependent effects. Additionally, the actual versus predicted values plot (Figure A1d) illustrates that the points closely align with the Y = X diagonal line, confirming a strong agreement between experimental and predicted values. Collectively, these results affirm that the regression model meets the underlying assumptions and demonstrates robust predictive capability.

#### 3.1.2. Response Surface and Contour Plots

**Figure 1:**
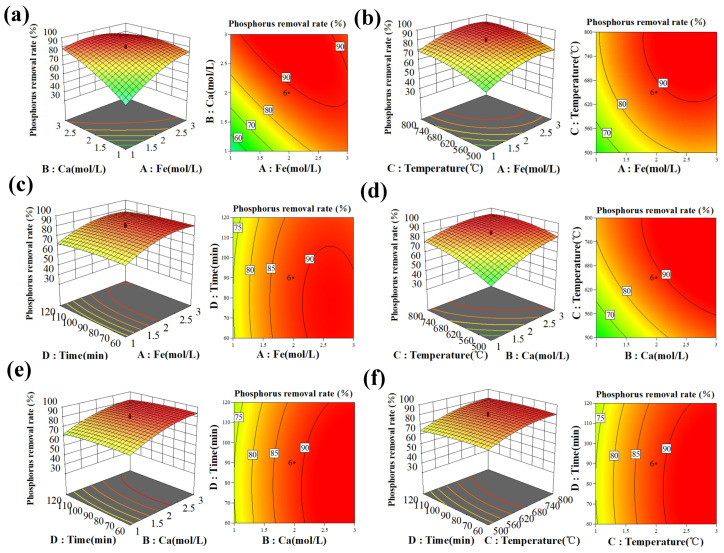
Response surface plots and contour plots of the effect: (**a**) Fe concentration and Ca concentration; (**b**) Fe concentration and pyrolysis temperature; (**c**) Fe concentration and pyrolysis time; (**d**) Ca concentration and pyrolysis temperature; (**e**) Ca concentration and pyrolysis time; (**f**) pyrolysis temperature and pyrolysis time.

The response surface for phosphorus adsorption interaction of the FCBC and the corresponding contour map (Figure 1) were generated based on the regression equation to elucidate the effects of independent variables and their interactions on the response value.

The results indicate that as the impregnation concentration of Fe and Ca materials increases, the phosphate removal rate also rises (Figure 1a). In comparison to Fe, the contour and response surface for Ca are denser and steeper, suggesting that Ca exerts a greater influence on the phosphate adsorption performance of FCBC. The phosphate removal rate increases with the impregnation concentration of Fe materials, reaching a maximum at approximately 2.5 mol/L (Figure 1b). Within the same range of Fe impregnation concentrations, the pyrolysis temperature exhibits a steeper gradient, while the pyrolysis time profile is relatively gentle (Figure 1b,c), indicating that pyrolysis temperature has a significantly greater impact than pyrolysis time. As the Ca impregnation concentration increases, the phosphate removal rate initially rises, stabilizes after reaching 2.0 mol/L, and then decreases slightly (Figure 1d,e), suggesting that the optimal molar ratio lies within the observed range. The interaction between pyrolysis temperature and pyrolysis time does not significantly affect phosphate adsorption (Figure 1f).

In conclusion, the relative significance of four independent variables in the preparation of fbbc for optimizing phosphate adsorption capacity is ranked as follows: Ca impregnation concentration > Fe impregnation concentration > pyrolysis temperature > pyrolysis time. This ranking aligns with the findings from the analysis of variance. Notably, the impact of pyrolysis temperature on the phosphate removal rate during material preparation is more pronounced than that of pyrolysis time, corroborating previous research results [30].

#### 3.1.3. Verification of Optimal Conditions

Through the analysis conducted using design-expert13, the optimal preparation process was identified as follows: the concentration of the Fe impregnation solution was 2.469 mol/L, the concentration of the Ca impregnation solution was 2.009 mol/L, the pyrolysis temperature was 716.998 °C, the pyrolysis time was 112.823 min, and the predicted phosphorus removal rate was 92%. Considering both experimental and practical factors, the parameters provided by the model were subsequently rounded: the concentration of the Fe impregnation solution was set at 2.5 mol/L, the concentration of the Ca impregnation solution at 2 mol/L, the pyrolysis temperature at 717 °C, and the pyrolysis time at 113 min. FCBC was prepared under these adjusted conditions. Three sets of parallel phosphorus adsorption experiments were conducted using simulated tail water under the same conditions as those in the response surface analysis experiment, and the experimental results were averaged.

**Figure 2:**
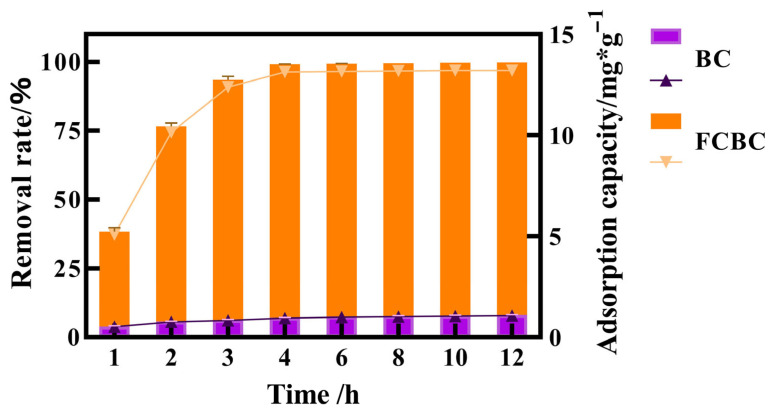
Comparison of phosphorus removal performance between biochar BC and optimized FCBC. Vertical bars and triangular curves represent removal efficiency and adsorbed phosphate capacity, respectively.

The results (Figure 2) indicate that BC exhibits minimal phosphorus removal capability, with a removal rate of less than 8% after 12 h of treatment. In contrast, FCBC demonstrated a high phosphorus removal efficiency, achieving a removal rate of 93.23% at 3 h, with a relative error of 1.23% compared to the predicted value, which was slightly higher than anticipated. Furthermore, the removal rate reached 99.28% at 4 h. These findings suggest that the response surface model is both reliable and effective for optimizing FCBC.

### 3.2. Characterization of Biochar

#### 3.2.1. SEM and EDS

**Figure 3:**
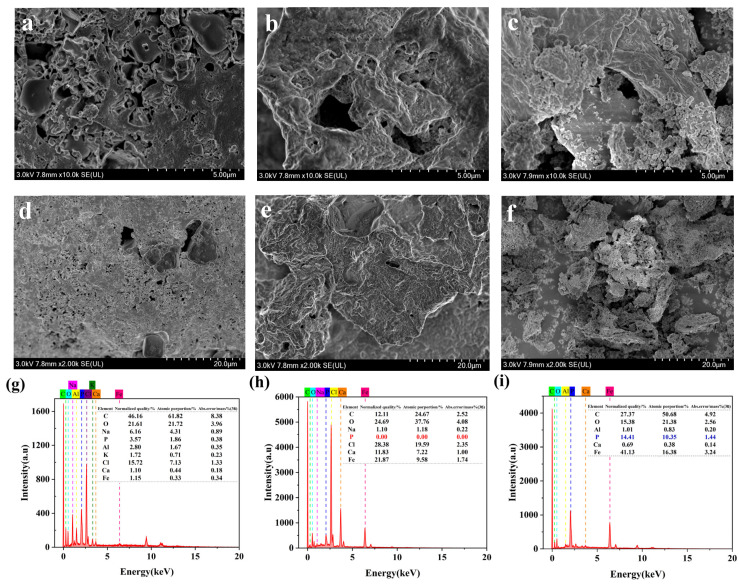
SEM images of (**a**) BC (5 µm); (**b**) FCBC (5 µm); (**c**) FCBC after adsorption (5 µm); (**d**) BC (20 µm); (**e**) FCBC (20 µm); (**f**) FCBC after adsorption (20 µm); and EDS spectra of (**g**) BC; (**h**) FCBC; (**i**) FCBC after adsorption.

As illustrated in Figure 3, the surface of biochar (BC) is devoid of particle load and predominantly exhibits a relatively smooth plane (Figure 3a,d). In contrast, following modification, the surface of functionalized biochar (FCBC) has undergone substantial changes, resulting in the near absence of a smooth plane and the emergence of numerous irregular, rough surfaces. A significant quantity of particles adheres to the biochar surface, forming relatively dense aggregates (Figure 3b,e). After the adsorption process (Figure 3c,f), the laminar flow structure of FCBC is markedly disrupted, leading to the formation of numerous irregular and rough particles on the surface, with these particles becoming agglomerated.

The EDS results of BC and FCBC showed that the normalized mass and atomic ratio of calcium and iron atoms in the modified biochar material were significantly improved (Figure 3g,h). After the adsorption process with FCBC, the phosphorus content increased significantly (Figure 3i), indicating the successful adsorption of P.

#### 3.2.2. Pore Structure

Table 4 shows the pore structure and specific surface area of BC and FCBC.

In comparison to BC, the average pore diameter and the most probable pore of FCBC are more than double those of BC. This finding aligns with the results obtained from scanning electron microscopy (SEM). However, the specific surface area of FCBC is nearly half that of BC, which may be attributed to the loading of iron and calcium. Furthermore, the pore volume of FCBC has experienced a slight increase. In summary, these results suggest that the binding of iron and calcium to the surface of BC biochar contributes to the observed increases in pore size and volume [33].

**Figure A2:**
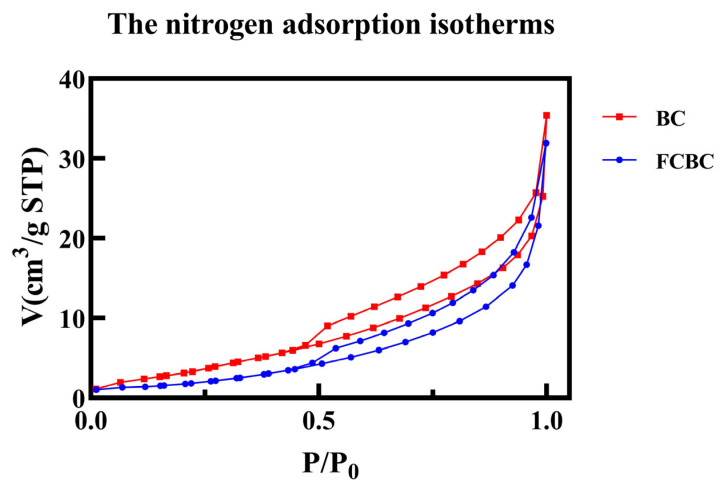
Nitrogen adsorption isotherm chart in BET detection.

The nitrogen adsorption–desorption isotherms for BC and FCBC are illustrated in Figure A2 (Appendix A). According to the IUPAC classification [34], both samples display type IV isotherms with H3 hysteresis loops, indicating the presence of mesoporous structures characterized by slit-shaped pores. BC demonstrates a relatively low nitrogen uptake of approximately 11.9 cm^3^∙g^−1^ at P/P_0_ ≈ 0.99, whereas FCBC shows a markedly higher uptake of around 31.9 cm^3^∙g^−1^, particularly exhibiting a sharp increase at P/P_0_ > 0.9. This increase suggests the formation of additional macropores or interparticle voids. This observation aligns with the enlarged average pore diameter of FCBC (22.77 nm) compared to BC (10.99 nm), as detailed in Table 4. Although the specific surface area of FCBC decreased following modification, the transition from a microporous/mesoporous to a macroporous/mesoporous structure facilitates phosphate diffusion and adsorption, thereby compensating for the reduction in surface area.

#### 3.2.3. XRD

**Figure 4:**
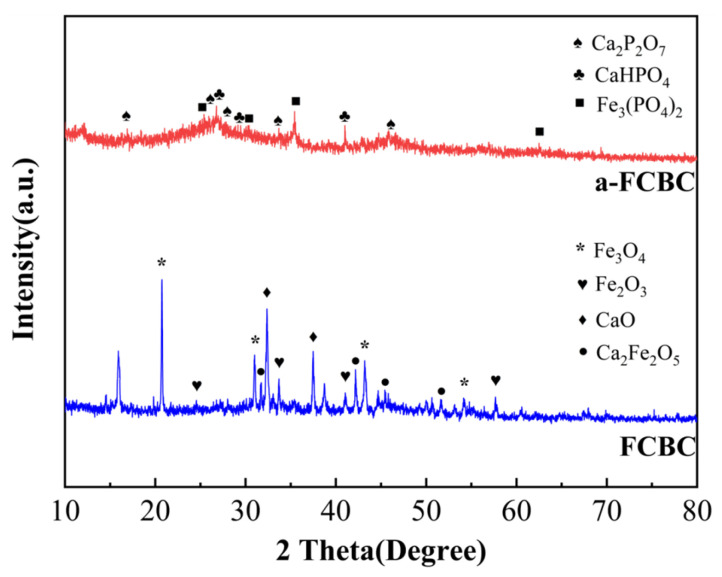
XRD spectra before and after FCBC adsorption. In this figure, a-FCBC is the detection result of the material after phosphorus adsorption.

The XRD patterns of FCBC before and after phosphate adsorption are presented in Figure 4. For pristine FCBC, diffraction peaks corresponding to Fe_3_O_4_, Fe_2_O_3_, CaO, and Ca_2_Fe_2_O_5_ were identified, confirming the successful incorporation of iron and calcium oxides into the biochar matrix during pyrolysis.

Following phosphate adsorption (denoted as a-FCBC), new diffraction peaks emerged, which were indexed to CaHPO_4_, Ca_2_P_2_O_7_, and Fe_3_(PO_4_)_2_. Concurrently, the intensities of the peaks associated with Fe_3_O_4_, Fe_2_O_3_, CaO, and Ca_2_Fe_2_O_5_ diminished relative to those in the pristine sample. Notably, Fe_3_(PO_4_)_2_ is recognized as a highly stable iron phosphate mineral [35,36].

#### 3.2.4. FTIR

**Figure 5:**
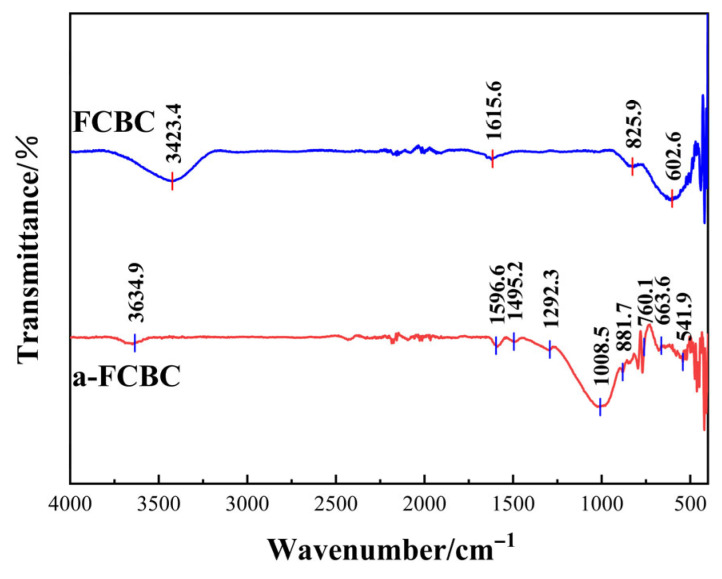
Fourier transform infrared (FTIR) spectra of FCBC before and after phosphate adsorption.

The FTIR spectra of FCBC before and after phosphate adsorption are presented in Figure 5. Prior to adsorption, the spectrum exhibits a broad O–H stretching band at 3423.4 cm^−1^ (surface hydroxyl groups and adsorbed water, including hydrated CaO) [37] and a sharp peak at 1615.6 cm^−1^ (C=C and O–H bending) [38]. Weak absorptions at 825.9 cm^−1^ and a broad band at 602.6 cm^−1^ are attributable to Fe–O stretching of iron oxides (Fe_3_O_4_, Fe_2_O_3_) and Fe–O–Ca vibrations from Ca_2_Fe_2_O_5_ [39], consistent with XRD-identified phases. After adsorption, the spectrum changes markedly. A strong peak at 1008.5 cm^−1^ (P-O stretching) confirms phosphate uptake [39,40]. Peaks at 881.7 cm^−1^ (HPO_4_^2−^) and 760 cm^−1^ (P–O–P symmetric stretching) provide direct evidence for CaHPO_4_ and Ca_2_P_2_O_7_ formation [40,41], corroborating XRD results. A weak peak at 1292.3 cm^−1^ may arise from residual carbon-oxygen or phosphate-related vibrations. The O–H stretching region shows significant changes: the 3423.4 cm^−1^ band diminishes, and a new feature at 3634.9 cm^−1^ appears, suggesting partial consumption of surface hydroxyls via ligand exchange [37]. The peak at 1615.6 cm^−1^ splits into two components at 1596.6 and 1495.2 cm^−1^, indicating Fe–OH and Ca–OH involvement in complexation or precipitation [38]. New absorptions at 663.6 and 541.9 cm^−1^, overlapping with the original Fe–O envelope, are attributed to Fe–O–P bond formation and O–P–O bending from Fe_3_(PO_4_)_2_ and calcium phosphate phases [41,42].

#### 3.2.5. XPS

**Figure A3:**
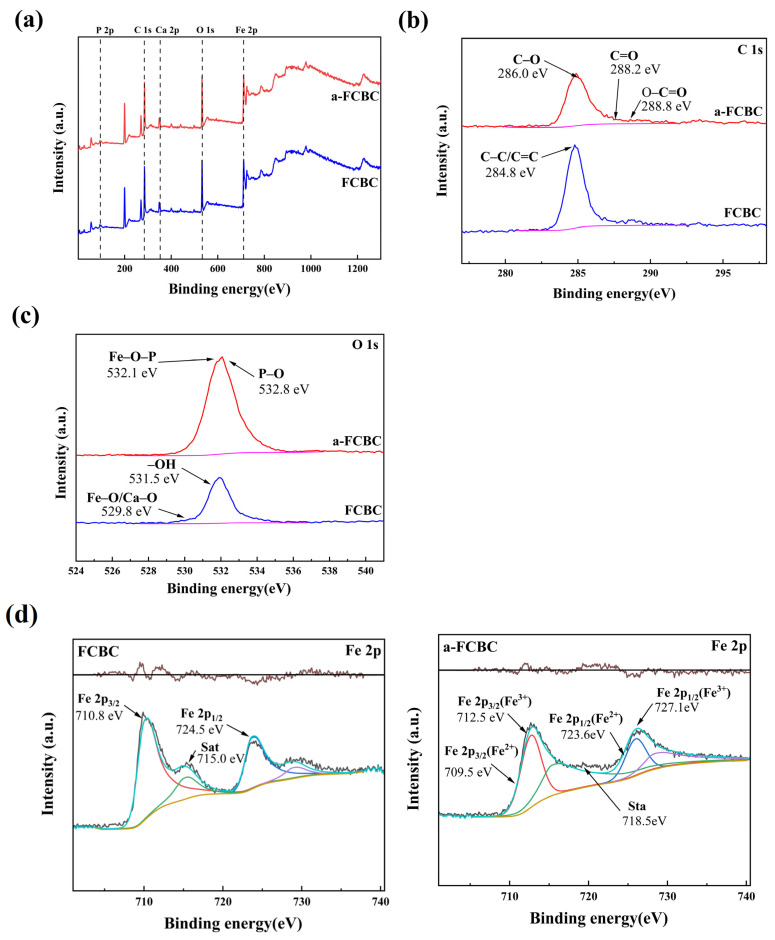

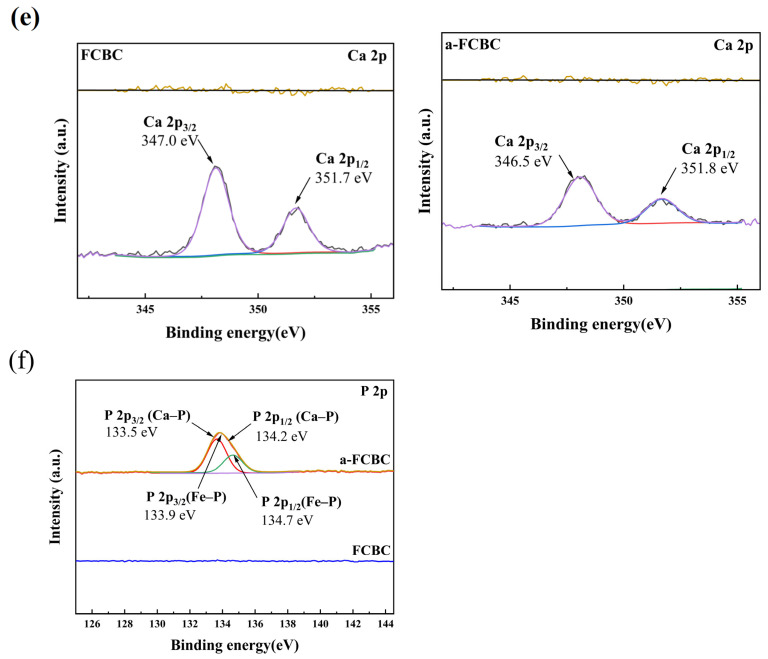
XPS detection results before and after FCBC adsorption, Survey spectrum (**a**), C1s spectrum (**b**), O 1s spectrum (**c**), Fe 2p spectrum (**d**), Ca 2p spectrum (**e**), P 2p spectrum (**f**). In this figure, a-FCBC is the detection result of the material after phosphorus adsorption. Peaks in different colors correspond to the deconvolution of the respective orbital spectra.

X-ray photoelectron spectroscopy (XPS) was employed to investigate the surface chemical composition and valence states of elements in FCBC before and after phosphate adsorption, and the results (Figure A3) are presented in Appendix A. The survey spectra (Figure A3a) confirm the presence of C, O, Fe, Ca, and, after adsorption, P. High-resolution spectra of C 1s, O 1s, Fe 2p, Ca 2p, and P 2p were further analyzed to elucidate the adsorption mechanisms.

The C 1s spectrum of pristine FCBC (Figure A3b) can be deconvoluted into three components: a dominant peak at 284.8 eV corresponding to sp^2^-hybridized carbon (C–C/C=C) of the biochar skeleton; a peak at 286.0 eV assigned to oxygen-containing functional groups (C–O, including hydroxyl and ether linkages); and a weak peak at 288.2 eV attributed to carbonyl groups (C=O) [43]. After phosphate adsorption, the intensity of the C–C/C=C peak decreases, while those of the C–O and C=O components increase significantly. This indicates that the biochar surface becomes partially covered by phosphate and metal oxides, and that the oxygen-containing functional groups participate in phosphate immobilization through hydrogen bonding or surface complexation, consistent with the FTIR observations.

The O 1s spectrum of FCBC before adsorption (Figure A3c) is deconvoluted into two main components: a peak at 529.8 eV attributed to lattice oxygen in metal oxides (Fe–O, Ca–O) and a peak at 531.5 eV corresponding to surface hydroxyl groups (–OH) [44]. Following phosphate adsorption, the O 1s envelope broadens significantly, and a new intense component emerges at 532.8 eV, characteristic of oxygen in phosphate groups (P–O). Concurrently, the intensity of the hydroxyl component decreases markedly, indicating substantial consumption of –OH groups during the adsorption process. These spectral changes provide direct evidence for a ligand exchange mechanism, wherein phosphate anions replace surface hydroxyl groups to form stable surface complexes, in full agreement with the FTIR results.

The Fe 2p spectrum of pristine FCBC (Figure A3d) exhibits the characteristic doublet of Fe 2p_3/2_ (710.8 eV) and Fe 2p_1/2_ (724.5 eV), with a spin–orbit splitting of 13.7 eV. Deconvolution of the Fe 2p_3/2_ peak reveals contributions from Fe^2+^ (≈709.8 eV) and Fe^3+^ (≈711.2 eV), and a distinct satellite feature at approximately 715 eV—characteristic of Fe^3+^ in octahedral coordination—confirms the presence of mixed-valence iron oxides such as magnetite (Fe_3_O_4_) and hematite (Fe_2_O_3_) [45], consistent with XRD analysis. After adsorption, the Fe 2p_3/2_ peak shifts to a higher binding energy (712.5 eV), accompanied by the appearance of a new satellite feature at 718.5 eV. These changes indicate oxidation of Fe^2+^ to Fe^3+^ by dissolved oxygen in seawater and the formation of Fe–O–P linkages, corresponding to the precipitation of iron phosphate (Fe_3_(PO_4_)_2_) [45], as identified by XRD and FTIR.

The Ca 2p spectrum of FCBC before adsorption (Figure A3e) displays a well-defined doublet with Ca 2p_3/2_ at 347.0 eV and Ca 2p_1/2_ at 351.7 eV (spin–orbit splitting of 4.7 eV), characteristic of Ca^2+^ in oxide environments such as CaO and Ca_2_Fe_2_O_5_ [46], corroborating the XRD results. Upon phosphate adsorption, the Ca 2p peaks broaden and shift slightly to lower binding energies (Ca 2p_3/2_ to 346.5 eV, Ca 2p_1/2_ to 351.8 eV), reflecting a change in the chemical environment due to the formation of insoluble calcium phosphate compounds, specifically CaHPO_4_ and Ca_2_P_2_O_7_ [46]. This finding is in excellent agreement with the appearance of characteristic diffraction peaks for these phases in the XRD patterns and the corresponding FTIR bands at 882 cm^−1^ and 760 cm^−1^.

The P 2p spectrum (Figure A3f) shows no detectable signal before adsorption, confirming the absence of phosphorus contamination in the pristine material. After adsorption, a well-resolved P 2p doublet appears, with a spin–orbit splitting of approximately 0.7 eV (slightly lower than the theoretical value of 0.84 eV due to peak overlap) and a peak area ratio of 2:1 for P 2p_3/2_ to P 2p_1/2_, consistent with the intrinsic properties of the P 2p orbital. Further deconvolution reveals two distinct doublets: one at 133.5 eV (P 2p_3/2_) and 134.2 eV (P 2p_1/2_), assigned to phosphate in calcium phosphate precipitates (CaHPO_4_, Ca_2_P_2_O_7_); and another at 133.9 eV (P 2p_3/2_) and 134.7 eV (P 2p_1/2_), corresponding to phosphate in iron phosphate complexes (Fe_3_(PO_4_)_2_ and inner-sphere Fe–O–P complexes). This provides direct evidence that phosphorus removal by FCBC involves two parallel pathways: calcium-mediated chemical precipitation and iron-based ligand exchange.

#### 3.2.6. Magnetic and Zeta Potential Properties

**Figure A4:**
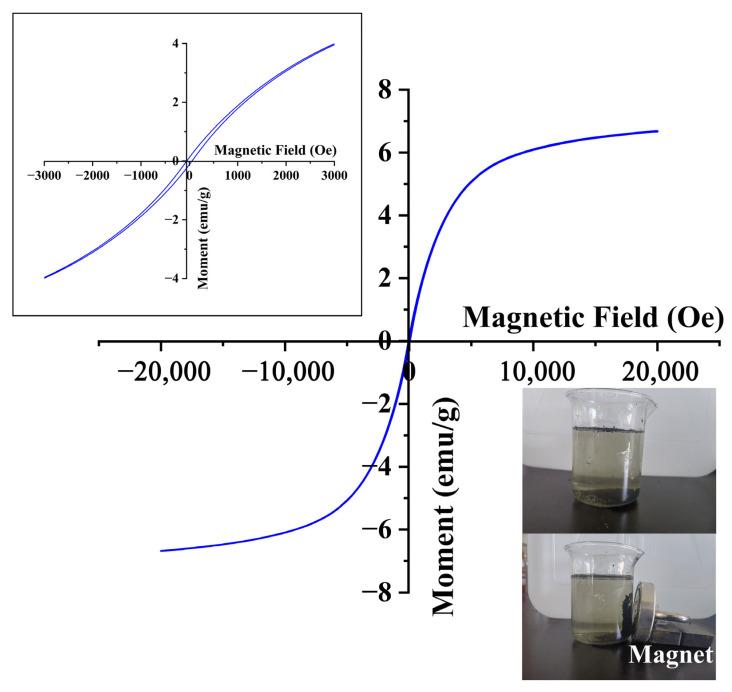
Magnetic characterization of FCBC. Main panel: full-range magnetization curve measured at 300 K. Inset (top left): enlarged view of the low-field region. A small separation between the forward and reverse scans is visible, which is likely due to a trapped magnetic field in the superconducting magnet and does not indicate intrinsic ferro- or ferrimagnetism. The photograph demonstrates rapid magnetic separation of FCBC particles using an external magnet.

The magnetic properties of FCBC were evaluated using a vibrating sample magnetometer (VSM). The magnetization curve is presented in Figure A4, with the main panel showing the full-range M–H curves of both forward and reverse scans. The sample exhibits a nearly linear increase in magnetization with applied field up to ±20,000 Oe, reaching a value of 6.68 emu∙g^−1^ at the maximum field, which is characteristic of paramagnetic behavior. A minor separation between the forward and reverse curves is observed near zero field (inset of Figure A4); however, such a feature is often caused by trapped magnetic field in the superconducting magnet rather than intrinsic magnetic hysteresis. Therefore, FCBC is considered to be essentially paramagnetic. Nevertheless, the saturation magnetization of 6.68 emu∙g^−1^ is sufficiently high to enable rapid separation of the adsorbent from the reaction system using an external magnetic field. As illustrated in the photograph of Figure A4, when a magnet was brought near the sample bottle, the FCBC particles were rapidly attracted and aggregated toward the magnet within seconds, demonstrating the good recoverability of FCBC.

**Figure A5:**
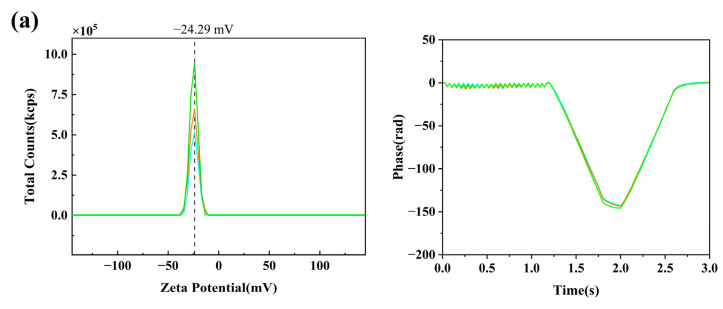

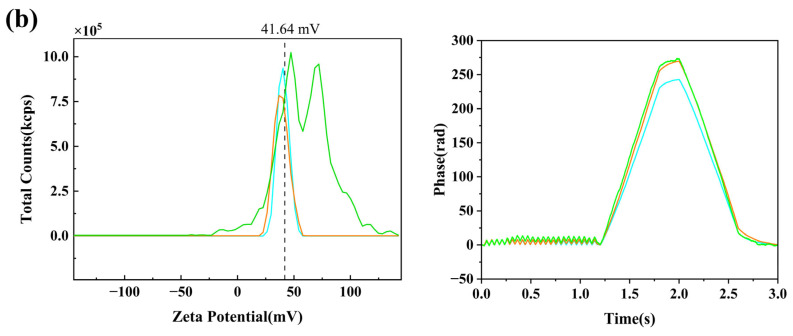
Zeta potential test results, BC results (**a**), FCBC results (**b**). In each panel, different colored curves represent superimposed results from multiple independent measurements.

Zeta potential energy measurements reveal (Figure A5, Table A3) that the average zeta potential of BC is −24.29 mV, indicating stability and a negative charge. In contrast, the average potential of modified FCBC increases to 41.64 mV, reflecting a significant positive charge. Furthermore, the quality factor (4.193) and the derived mean count rate (4918 kcps) of the modified samples are markedly higher than those of the unmodified samples, suggesting enhanced test signal strength and improved data reliability for the modified samples.

### 3.3. Adsorption

**Figure 6:**
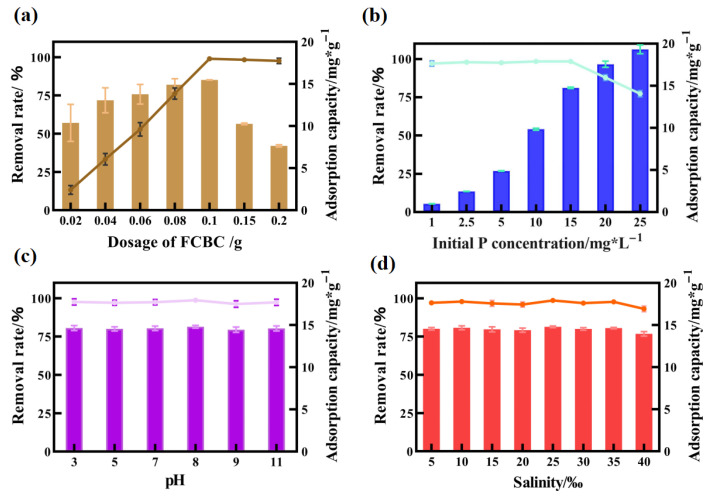
Effects of (**a**) FCBC dosage, (**b**) initial phosphate concentrations, (**c**) pH, and (**d**) salinity on phosphate adsorption of FCBC. Vertical bars represent adsorbed phosphate capacity; triangular curves represent removal efficiency.

The effects of material dosage, initial phosphorus concentration, pH and salinity on the adsorption process were systematically investigated (Figure 6).

#### 3.3.1. Dosage

The results (Figure 6a) indicated that increasing the dosage of FCBC enhanced both the available adsorption sites and the total phosphate adsorption capacity. However, as the dosage continued to rise, the unit adsorption capacity (*q_e_*) experienced a significant decline. This decrease occurred because the adsorption sites were fully utilized at lower dosages, while the utilization rate diminished due to the presence of excess sites at higher dosages. At a dosage of 0.1 g per 100 mL, the phosphorus removal rate approached 99%, with an optimal dosage determined to be 1.0 g/L based on a comprehensive assessment of removal efficiency and economic considerations.

#### 3.3.2. Initial Phosphate Concentration

FCBC demonstrated effective adsorption adaptability across an initial phosphorus concentration range of 1 to 25 mg/L (Figure 6b). Within the 1 to 15 mg/L range, the removal rate exceeded 97%. Although the unit adsorption capacity increased with rising concentration, the overall removal rate declined due to the saturation of certain adsorption sites.

#### 3.3.3. pH

The phosphorus removal rate of FCBC across the pH range of 3 to 11 is remarkably consistent (Figure 6c), with both rates exceeding 96%. This indicates that FCBC retains a high adsorption capacity over a broad pH spectrum. The extensive pH adaptability is attributed to the Fe/Ca synergistic mechanism [47,48]. Under acidic conditions, electrostatic adsorption and ligand exchange predominantly govern the behavior of iron oxides. In contrast, calcium phosphate precipitation, resulting from the interaction between calcium ions and phosphate radicals, prevails in moderately alkaline conditions. Additionally, the local pH buffering effect of the material’s surface microenvironment further enhances its stability throughout the entire pH range [48].

#### 3.3.4. Salinity

The phosphorus removal rates of FCBC across a salinity range of 5 to 40‰ are consistently high, exceeding 97% (Figure 6d), which indicates a broad adaptability to salinity. This remarkable salt tolerance primarily results from the calcium-based precipitation mechanism’s insensitivity to competitive anions such as Cl^−^ and SO_4_^2−^, as well as the facilitation of the precipitation reaction by background Ca^2+^ in seawater [49]. Additionally, the surface complexation of iron oxides exhibits a degree of competitiveness against ions, which collectively contributes to the efficient phosphorus removal performance of FCBC in high-salinity environments [50,51].

### 3.4. Adsorption Kinetics

**Figure 7:**
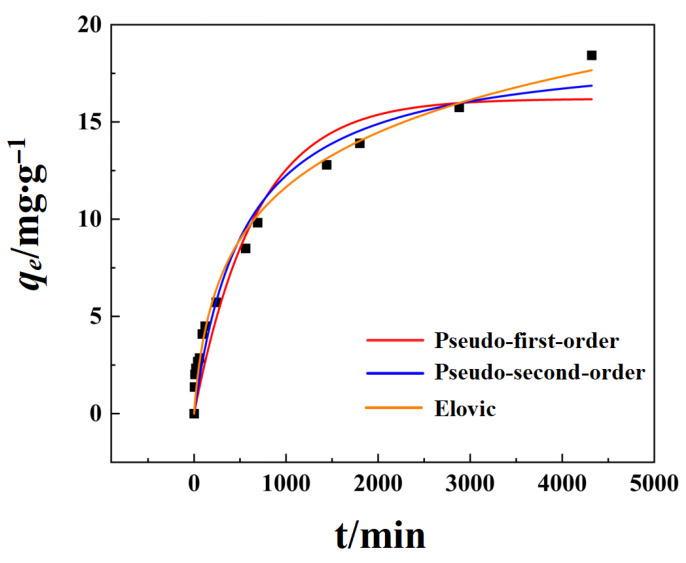
Pseudo-first-order kinetic model, Pseudo-second-order kinetic model and Elovich dynamic models of FCBC. Black squares represent the raw data.

The adsorption kinetics of phosphate on FCBC were examined using quasi-first-order, quasi-second-order, and Elovich models (Figure 7) to elucidate the relationship between adsorption kinetics and contact time. The majority of phosphate adsorption on FCBC occurred within the initial 690 min, after which the adsorption rate progressively diminished as the reaction time increased.

The results are presented in Table 5. The adsorption process aligns more closely with the quasi-second-order kinetic model, as evidenced by a significantly higher correlation coefficient (R^2^ > 0.98) compared to the quasi-first-order kinetic model. This finding suggests that the adsorption of phosphate by FCBC is primarily governed by chemical adsorption [52]. Furthermore, the Elovich model exhibits the highest fitting correlation coefficient, indicating that the phosphate adsorption process on FCBC is a heterogeneous chemical adsorption phenomenon occurring on uneven surfaces [53]. This observation further supports the conclusion that the FCBC surface possesses a multi-layered concave-convex structure, which is consistent with the results of SEM (Figure 3).

### 3.5. Adsorption Isotherm

**Figure 8:**
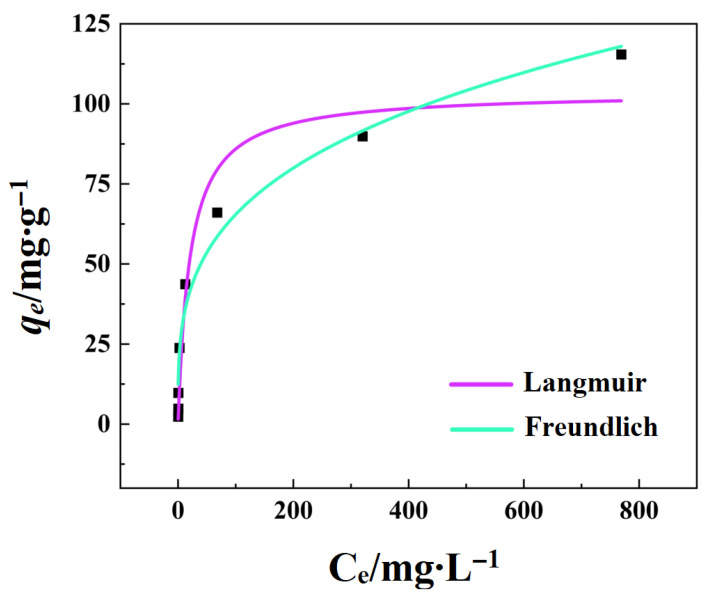
Isothermal adsorption fitting curve of FCBC.Black squares represent the raw data.

Langmuir and Freundlich isothermal models were used to fit the results of phosphate adsorption by FCBC (Figure 8), and the relevant fitting parameters are shown in Table 6.

The correlation coefficient of the Freundlich model after the second iteration (R^2^ = 0.971) was significantly higher than that of the Langmuir model (R^2^ = 0.935), indicating that the adsorption process aligns more closely with the Freundlich model. This finding suggests that the modified FCBC exhibits considerable heterogeneity, characterized by a continuous energy distribution of its active sites. Consequently, the phosphate adsorption process is better represented by the Freundlich model, which accounts for multi-layer adsorption and heterogeneous surface adsorption, in contrast to the uniform energy sites posited by the Langmuir model [54]. Furthermore, the Freundlich constant 1/*n* is substantially less than 1, demonstrating that FCBC possesses a notable advantage in phosphate adsorption.

## 4. Discussion

### 4.1. Optimization of FCBC Preparation

The Box–Behnken design coupled with response surface methodology successfully identified the optimal preparation conditions for FCBC and elucidated the relative significance of the four factors examined. Calcium concentration emerged as the most influential variable (F = 221.18, *p* < 0.0001), underscoring the dominant role of calcium-mediated precipitation in the weakly alkaline (pH 7.5–8.0) simulated mariculture tail water. This pathway exhibits inherent resilience to competitive anions (Cl^−^, SO_4_^2−^) and is further potentiated by the elevated background Ca^2+^ concentration (~400 mg·L^−1^) characteristic of seawater [50]. Although iron concentration exerted a secondary influence on overall removal efficiency, its contribution to accelerating initial adsorption kinetics was substantial, as evidenced by rapid phosphorus uptake within the first 3 h and excellent conformity to the pseudo-second-order kinetic model (R^2^ = 0.987). The highly significant AB interaction term (*p* < 0.0001) substantiates the synergistic interplay between Fe and Ca: iron provides abundant surface hydroxyl groups for rapid ligand exchange, while calcium ensures long-term stability through chemical precipitation. Pyrolysis temperature (C, *p* < 0.0001) also emerged as a critical determinant, as elevated temperatures (optimized at 717 °C) promote graphitization of the carbon skeleton and facilitate the transformation of Fe and Ca precursors into thermally stable oxides (Fe_3_O_4_, CaO, Ca_2_Fe_2_O_5_) that resist leaching in saline media [55,56].

In contrast, pyrolysis time (D) exerted a non-significant effect (*p* = 0.0856), suggesting that within the evaluated range (60–120 min) the pyrolysis process reaches completion, and prolonged duration does not substantially alter the final material properties. These findings collectively validate the appropriateness of the BBD approach and confirm the suitability of RSM for optimizing a multi-variable system exhibiting smooth, continuous response behavior [30,31].

### 4.2. Structural and Surface Chemical Evolution

The incorporation of Fe and Ca oxides profoundly reconfigured the textural properties of FCBC. Compared to pristine BC, the specific surface area of FCBC decreased from 13.97 to 7.21 m^2^·g^−1^, while the average pore diameter increased from 10.99 to 22.77 nm and the pore volume exhibited a modest increase (Table 4). Although the specific surface area decreased upon modification, this was accompanied by a substantial increase in average pore diameter. This apparent trade-off can be rationalized by the loading of metal oxide particles that partially block or occlude micropores, while simultaneously generating larger mesopores and macropores through pore-wall collapse and oxide aggregation during high-temperature pyrolysis [33,57]. The nitrogen adsorption–desorption isotherms (Figure A1) confirm a type IV profile with H3 hysteresis, characteristic of slit-shaped mesopores, and the pronounced uptake increase at P/P_0_ > 0.9 for FCBC indicates the formation of additional macropores or interparticle voids.

Critically, the observed changes should not be interpreted as an optimized expansion of the pore network in the sense of preserving microporosity. Rather, they reflect a structural reorganization in which a portion of the biochar’s inherent microporosity was sacrificed due to physical blockage and partial collapse caused by the growth of Fe/Ca oxide particles during pyrolysis [57,58]. This interpretation acknowledges that the reduction in specific surface area represents a compromise inherent to the modification process.

Nevertheless, the enlarged pore channels substantially lower mass transfer resistance and facilitate phosphate diffusion toward the active sites. This is particularly advantageous given the ionic diameter of phosphate (~0.34 nm), and the fact that the channels are now predominantly in the mesoporous/macroporous range further enhances this benefit. Thus, while the loss of microporosity is an unavoidable consequence of metal oxide loading, the resulting structural transformation enhances the accessibility of active Fe/Ca sites and partially compensates for the surface area loss. This situation has also occurred in the research of other metal oxide modified biochars [57,58].

### 4.3. Comprehensive Adsorption Mechanism

**Figure 9:**
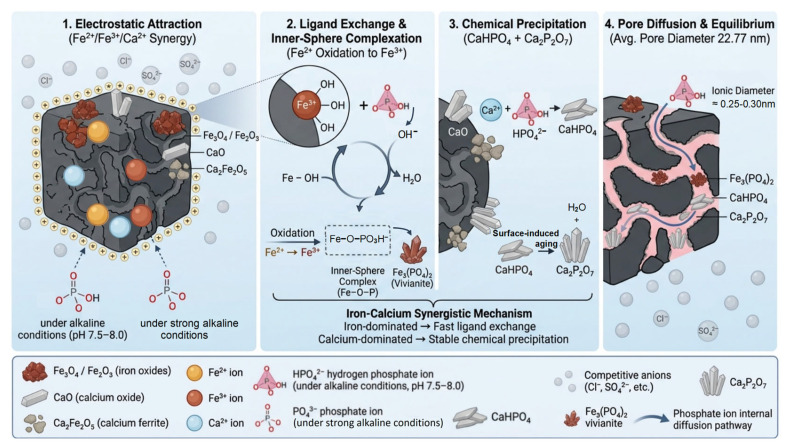
Process and mechanism of phosphorus adsorption by FCBC.

The combination of XRD, FTIR, XPS, and zeta potential measurements provides a comprehensive understanding of the multi-stage adsorption mechanism by which FCBC removes phosphate from mariculture tail water. Based on these complementary characterization techniques, the phosphorus removal process can be delineated into four sequential and synergistic stages: electrostatic attraction, ligand exchange (inner-sphere complexation), chemical precipitation, and pore diffusion. Together, these stages form an integrated mechanism that explains the rapid kinetics, high capacity, and exceptional environmental adaptability of FCBC. The simplified process and mechanisms are illustrated in Figure 9.

The initial stage is characterized by rapid electrostatic attraction. The zeta potential of FCBC (+41.6 mV) confirms a strongly positively charged surface, which originates from protonation of Fe/Ca hydroxyl groups (≡M–OH + H^+^ → ≡M–OH_2_^+^) under near-neutral conditions [7,59]. In mariculture tail water (pH 7.5–8.0), phosphorus predominantly exists as negatively charged species, particularly HPO_4_^2−^ [60]. This positive surface charge facilitates the swift capture of phosphate ions from the bulk solution through electrostatic attraction, forming an initial adsorption layer that is crucial for subsequent deep adsorption. This stage is particularly important during the early phase of the adsorption process and serves as a critical prerequisite for FCBC to achieve rapid phosphorus removal within the first 3 h of contact time [61].

Following electrostatic attraction, the system transitions into a ligand exchange stage. During this phase, phosphate ions undergo ligand exchange with surface hydroxyl groups on iron oxides. FTIR spectra provide compelling evidence for this mechanism: the broad O–H stretching band at 3423.4 cm^−1^ diminishes in intensity after adsorption, and a new feature at 3634.9 cm^−1^ emerges, indicating consumption of surface hydroxyl groups [37]. Concomitantly, the Fe–O band at 602.6 cm^−1^ undergoes profile changes, and new absorptions emerge at 663.6 and 541.9 cm^−1^, which are attributed to Fe–O–P bond formation [41,42]. These spectral changes indicate that phosphate radicals replace –OH groups in Fe–OH, leading to the formation of stable inner-sphere complexes. XPS further corroborates this mechanism: the Fe 2p_3/2_ peak shifts from 710.8 eV (characteristic of mixed Fe^2+^/Fe^3+^ in Fe_3_O_4_ and Fe_2_O_3_) to 712.5 eV after adsorption, consistent with oxidation of Fe^2+^ to Fe^3+^ and the development of Fe–O–P linkages [45]. The oxidation of Fe^2+^ enhances ligand exchange capacity, as Fe^3+^ exhibits higher electronegativity and a significantly greater complexation constant with phosphate (log K = 23.8) compared to Fe^2+^ (log K = 15.1) [62]. This mechanism predominates under acidic to neutral conditions (pH 3–7) [63], elucidating the consistent phosphorus removal efficacy of FCBC across a broad acidic range.

The third stage is governed by chemical precipitation, with calcium playing a complementary yet distinct role. Under the weakly alkaline conditions of mariculture tail water (pH 7.5–8.0), Ca^2+^ released from the FCBC surface reacts with HPO_4_^2−^ to form calcium hydrogen phosphate. The reaction is shown by Equation (9) [64]. The formation of CaHPO_4_ is substantiated by XRD, which reveals characteristic diffraction peaks indexed to this phase after adsorption (Figure 4) [35,36]. The FTIR band at 881.7 cm^−1^ is assigned to the HPO_4_^2−^ group, providing complementary vibrational spectroscopic evidence [40,41]. Additionally, XPS analysis of the Ca 2p region reveals a shift from 347.0 eV in pristine FCBC (characteristic of CaO and Ca_2_Fe_2_O_5_) to 346.5 eV after adsorption, consistent with the transformation of calcium from an oxide environment to a phosphate-associated chemical state [48]. Additionally, the high background concentration of Ca^2+^ in seawater (approximately 400 mg·L^−1^) serves as an additional source of calcium that promotes continuous precipitation, contributing to the sustained high removal efficiency of FCBC even at elevated salinity levels [53,65]. Notably, XRD also detects the presence of Ca_2_P_2_O_7_ (calcium pyrophosphate) alongside CaHPO_4_. This phase is not a direct solution precipitate but rather a product of surface-induced aging [66]. Within the confined microenvironment of the FCBC porous structure, which is characterized by locally elevated concentrations of Ca^2+^ and adsorbed phosphate, adjacent CaHPO_4_ units undergo dehydration and condensation over extended contact periods. The reaction is shown by Equation (10) [67]. The formation of Ca_2_P_2_O_7_ is supported by FTIR, where the band at 760 cm^−1^ is assigned to the P–O–P symmetric stretching vibration characteristic of pyrophosphate groups. This transformation is time-dependent, aligning with the adsorption kinetics (Figure 7), which exhibit a sustained uptake phase beyond the initial rapid adsorption. The coexistence of CaHPO_4_ (kinetically favored) and Ca_2_P_2_O_7_ (formed via slow aging) reflects an intermediate state of this surface-induced phase transformation, providing direct evidence for a two-stage calcium-mediated immobilization mechanism [66].

(9) $${\mathrm{Ca}}^{2+}+{\mathrm{HPO}}_{4}^{2-}\to \;{\mathrm{CaHPO}}_{4}↓$$

(10) $$2{CaHPO}_{4}\to \;{\mathrm{Ca}}_{2}P_{2}O_{7}↓+H_{2}O$$

The final stage involves pore diffusion and the attainment of adsorption equilibrium. Concurrently with surface reactions, phosphate ions diffuse into the reorganized porous network of FCBC. The nitrogen adsorption–desorption isotherms (Figure A2) and pore structure parameters (Table 4) reveal that FCBC possesses enlarged pore channels, with an average pore diameter of 22.77 nm (predominantly in the mesoporous/macroporous range). These enlarged channels substantially lower mass transfer resistance and facilitate the inward diffusion of phosphate ions, allowing them to access active sites located deeper within the biochar matrix. Given that the ionic diameter of phosphate is approximately 0.25–0.30 nm, the mesoporous/macroporous architecture poses minimal diffusional constraint. This physical transport mechanism complements the chemical interactions described in the preceding stages and ensures that the entire porous network contributes to the overall adsorption capacity. As surface sites approach saturation, the system reaches adsorption–desorption equilibrium, characterized by a near-zero net adsorption rate [68].

The four stages operate in a concerted and synergistic manner: electrostatic attraction rapidly concentrates phosphate at the positively charged surface, ligand exchange anchors it onto iron sites through strong inner-sphere bonding, chemical precipitation with calcium produces highly insoluble phases that ensure long-term immobilization, and pore diffusion guarantees full utilization of the material’s internal capacity. This multi-mechanistic approach, confirmed by the convergence of XRD, FTIR, and XPS evidence, explains the superior performance of FCBC under the challenging conditions of high-salinity, weakly alkaline mariculture tail water. The synergy between iron and calcium is particularly critical: iron provides rapid chemisorption kinetics, while calcium confers stable, long-term immobilization even in the presence of competitive anions and under varying pH conditions.

The four stages operate in a concerted and synergistic manner. The XPS results, in full agreement with XRD and FTIR analyses, collectively establish a multi-mechanistic adsorption process: (i) ligand exchange between phosphate and surface hydroxyl groups on iron oxides, forming inner-sphere Fe–O–P complexes and Fe_3_(PO_4_)_2_ precipitates; (ii) chemical precipitation of Ca^2+^ with phosphate to form insoluble CaHPO_4_ and Ca_2_P_2_O_7_; and (iii) auxiliary involvement of biochar surface oxygen-containing functional groups (C–O, C=O) through hydrogen bonding or surface complexation. The convergence of evidence from three complementary techniques provides a robust and comprehensive understanding of the superior phosphate removal performance of FCBC under the challenging conditions of high-salinity, weakly alkaline mariculture tail water. The synergy between iron and calcium is particularly critical: iron provides rapid chemisorption kinetics, while calcium confers stable, long-term immobilization even in the presence of competitive anions and under varying pH conditions.

### 4.4. Exceptional Adaptability and Comparative Advantage

FCBC demonstrates outstanding performance across a wide pH range (3–11) and high salinities (5–40‰), consistently achieving >97% phosphorus removal. This broad adaptability stems from the complementary roles of iron and calcium. Under acidic to neutral conditions (pH 3–7), ligand exchange with iron oxides dominates, as the surface hydroxyl groups remain protonated and readily undergo exchange with phosphate. Under alkaline conditions (pH 7–11), calcium-mediated precipitation becomes the primary mechanism; the formation of CaHPO_4_ and Ca_2_P_2_O_7_ is insensitive to OH^−^ competition and is even enhanced by the elevated Ca^2+^ background in seawater [35,36]. Moreover, the local pH buffering effect, involving OH^−^ release during ligand exchange under acidic conditions and OH^−^ consumption during precipitation under alkaline conditions, helps stabilize the microenvironment around active sites [69].

In high-salinity environments, conventional iron-modified biochars suffer from severe competitive anion interference and active component leaching, leading to drastic efficiency reductions [19,20]. Calcium-only modifications, while stable, exhibit sluggish kinetics [21,22]. FCBC overcomes these limitations: the inner-sphere complexes formed with iron are highly specific and resistant to ionic competition [70], and the calcium phosphate precipitation pathway is unaffected by Cl^−^ or SO_4_^2−^ because these anions do not form less soluble compounds with Ca^2+^ [50]. Consequently, FCBC can sustain its removal efficiency at a salinity of 40 ‰, surpassing that of many conventional metal-modified biochars. For instance, single-metal iron biochar generally demonstrates a removal rate of ≤80% at salinities exceeding 30 ‰ [20], whereas calcium-based adsorbents typically necessitate longer contact times [22]. The synergistic effect realized in FCBC signifies a notable advancement in phosphorus removal technology for the treatment of high salinity and weakly alkaline seawater aquaculture effluent.

### 4.5. Limitations and Future Perspectives

Despite the promising results, several limitations merit acknowledgment:

Although the enlarged pore size facilitates diffusion, the concomitant reduction in specific surface area implies that some microporosity, and thus potential adsorption capacity, was sacrificed. Future work could explore strategies to better preserve microporosity, such as optimizing the Fe/Ca loading ratio or employing templating agents during pyrolysis [70].

In the preliminary experiments, iron–calcium-modified microalgae biochar was evaluated against biochar derived from various raw materials, including corn straw, wheat straw, and rapeseed straw, under identical conditions to simulate phosphorus removal in seawater aquaculture wastewater. Among these, iron–calcium-modified microalgae biochar demonstrated superior removal performance. However, the preparation conditions for FCBC have not been optimized through response surface analysis in these initial experiments. To maintain the rigor of the research, the findings from this segment of the study were not included in the current report. Given the existing experimental conditions and time limitations, we will proceed to compare the phosphorus removal efficacy of FCBC produced under optimized preparation conditions with that of biochar materials derived from other raw materials, prepared under the same conditions.

Furthermore, although the RSM model exhibited strong predictive capability, the optimization relied on a limited set of factors, specifically Fe/Ca concentration, pyrolysis temperature, and time. Other potentially influential parameters, including heating rate, impregnation time, and biomass particle size, were not systematically examined and may further impact phosphorus removal performance. Future investigations could adopt a more comprehensive experimental design that incorporates these additional factors.

Fourthly, although FCBC demonstrated remarkable phosphorus removal in batch experiments, its efficacy in continuous-flow systems more reflective of practical mariculture tail water treatment has yet to be assessed. Further investigation is necessary regarding factors such as hydraulic retention time, flow rate, and long-term stability under dynamic conditions. Moreover, it is essential to evaluate the potential leaching of Fe and Ca ions during extended operation to ensure environmental safety.

Finally, although FCBC exhibits paramagnetic behavior with a saturation magnetization of 6.68 emu·g^−1^, which enables efficient magnetic separation, the magnetic recovery efficiency may decline after repeated use, suggesting that the material’s magnetic responsiveness could be partially compromised during extended operation. Developing a protective coating, such as calcium alginate encapsulation [71,72], could enhance recoverability and minimize metal ion leaching. The regeneration and reusability of spent FCBC, as well as its possible application as a slow-release phosphate fertilizer, warrant further exploration to align with circular economy principles [73,74]. Addressing these aspects in future research will facilitate the translation of FCBC toward practical implementation in mariculture tail water treatment.

## 5. Conclusions

In this study, a novel iron–calcium co-modified Chlorella biochar (FCBC) was successfully synthesized and optimized using Box–Behnken response surface methodology, achieving optimal preparation conditions of Fe 2.5 mol·L^−1^, Ca 2.0 mol·L^−1^, 717 °C, and 113 min, which yielded a phosphorus removal rate of 93.23% within 3 h. Comprehensive characterization confirmed the successful loading of Fe/Ca oxides, endowing FCBC with a positively charged surface, good magnetic responsiveness (saturation magnetization of 6.68 emu·g^−1^), and an enlarged pore architecture. The material demonstrated outstanding adaptability, maintaining >97% phosphorus removal across pH 3–11 and salinities up to 40‰. Adsorption kinetics followed the pseudo-second-order model, and equilibrium data fitted the Freundlich isotherm, indicating heterogeneous chemisorption. The mechanism involves a synergistic four-stage process: electrostatic attraction, ligand exchange forming Fe–O–P complexes, chemical precipitation of CaHPO_4_ and Ca_2_P_2_O_7_, and pore diffusion. The iron-calcium synergy overcomes the limitations of conventional single-metal modified biochars, offering rapid kinetics and long-term stability, while its magnetic properties facilitate easy recovery. This work provides a viable and sustainable adsorbent for phosphorus removal in mariculture tail water treatment, with the phosphorus-loaded material showing promise as a slow-release fertilizer in alignment with circular economy principles.

## 6. Patents

The FCBC material in this article has applied for a patent with the State Intellectual Property Office (application No.: 2025115482880), and is currently in the review stage.

## Figures and Tables

**Table 1:** Response surface design factor level.

Level	A	B	C	D
	Fe Concentration/(mol·L^−1^)	Ca Concentration/(mol·L^−1^)	Pyrolysis Temperature/°C	Pyrolysis Time/min
−1	1	1	500	60
0	2	2	650	90
1	3	3	800	120

**Table 2:** Details of representation methods.

Instrument Type	Research Objective
Field emission scanning electron microscope (SEM-EDS, Quanta FEG 450, FEI Company, Hillsboro, OR, USA)	Surface morphology and elemental distribution.
Specific surface area analyzer (BET, TriStar II Plus 3030, Micromeritics Instrument Corporation, Norcross, GA, USA)	Specific surface area, pore volume, and pore size distribution.
X-ray diffraction spectrum (XRD, Bruker D8 advance, Bruker AXS GmbH, Karlsruhe, Germany)	Crystalline phase identification.
X-ray photoelectron spectrometer (XPS, Shimadzu Kratos Axis Supra ^TM^, Kratos Analytical Ltd., Manchester, UK)	Surface chemical composition, elemental binding energy, and chemical states before and after adsorption.
Fourier transform infrared spectrometer (FTIR, Thermo Fisher Nicolet iS20, Thermo Fisher Scientific, Waltham, MA, USA)	Surface functional groups and chemical bonding.
Vibrating sample magnetometer (VSM, Quantum Design DynaCool, Quantum Design, San Diego, CA, USA)	Saturation magnetization and magnetic separability.
Zeta potential meter (Zeta potential, Marvin S90, Malvern Panalytical Ltd., Malvern, UK)	Surface charge characteristics.

**Table 3:** Analysis of variance results of quadratic regression equation.

Variance Source	Sun of Squares	Df	Mean Square	F Value	*p*-Value Prod > F
Model	7746.26	14	553.30	57.13	<0.0001
A	1407.62	1	1407.62	145.34	<0.0001
B	2142.15	1	1993.66	221.18	<0.0001
C	1202.06	1	1202.06	124.12	<0.0001
D	32.79	1	32.79	3.39	0.0856
AB	1111.48	1	1111.48	114.76	<0.0001
AC	21.23	1	21.23	2.19	0.1594
AD	1.37	1	1.37	0.1416	0.7120
BC	203.99	1	203.99	21.06	0.0004
BD	0.7379	1	0.7379	0.0762	0.7863
CD	12.13	1	12.13	1.25	0.2807
A^2^	1046.86	1	1046.86	108.09	<0.0001
B^2^	527.49	1	527.49	54.47	<0.0001
C^2^	460.03	1	460.03	47.50	<0.0001
D^2^	55.21	1	55.21	5.70	0.0306
Residual	145.27	15	9.68		
Lack of fit	126.26	10	12.63	3.32	0.0987
Pure error	19.01	5	3.80		
Cor Total	7891.54	29			

**Table 4:** Pore structure parameters of BC and FCBC materials.

Sample	Specific Surface Area/(m^2^∙g^−1^)	Average Pore Diameter/nm	Pore Volume/(cm^3^∙g^−1^)	Most Probable Pore Diameter/nm
BC	13.97	10.99	0.038	2.45
FCBC	7.21	22.77	0.042	4.93

**Table 5:** Reaction kinetics fitting parameters.

Models	Pseudo-First-Order			Pseudo-Second-Order			Elovich		
	*q_e_*/(mg·g^−1^)	*k*_1_/h^−1^	R^2^	*q_e_*/(mg·g^−1^)	*k*_2_	R^2^	*α*/(mg·g^−1^·h^−1^)	*β*/(g·mg^−1^)	R^2^
FCBC	16.192	0.00149	0.929	19.029	9.48·10^−5^	0.987	0.0616	0.235	0.988

**Table 6:** Fitted parameters of adsorption isotherm models.

Models	Langmuir			Freundlich		
	*q_m_*/(mg·g^−1^)	*K*_L_/h^−1^	R^2^	*K*_F_/(L^1/*n*^·mg^1−1/*n*^·g^−1^)	1/*n*	R^2^
FCBC	103.67	0.0483	0.935	17.329	0.289	0.971

## Data Availability

The original contributions presented in this study are included in the article. Further inquiries can be directed to the corresponding author.

## Appendix A

### Appendix A.1. Table Supplement of Response Surface Optimization Experiment

**Table A1:** Design and Results of Response Surface Optimization Scheme.

Experiment Number	A	B	C	D	Phosphorus Removal Rate/%
1	−1	−1	−1	−1	35.080
2	1	−1	−1	−1	73.033
3	−1	1	−1	−1	83.470
4	1	1	−1	−1	80.402
5	−1	−1	1	−1	60.564
6	1	−1	1	−1	88.544
7	−1	1	1	−1	90.201
8	1	1	1	−1	86.517
9	−1	−1	−1	1	32.403
10	1	−1	−1	1	66.926
11	−1	1	−1	1	78.570
12	1	1	−1	1	75.215
13	−1	−1	1	1	62.322
14	1	−1	1	1	85.382
15	−1	1	1	1	86.456
16	1	1	1	1	86.725
17	−2	0	0	0	45.291
18	2	0	0	0	80.353
19	0	−2	0	0	54.132
20	0	2	0	0	85.852
21	0	0	−2	0	59.093
22	0	0	2	0	83.212
23	0	0	0	−2	82.920
24	0	0	0	2	80.800
25	0	0	0	0	87.405
26	0	0	0	0	90.300
27	0	0	0	0	91.549
28	0	0	0	0	87.434
29	0	0	0	0	89.1511
30	0	0	0	0	86.495

**Table A2:** Reliability analysis of regression equation.

Project	Result
Related R^2^	0.9816
Adjusted R^2^	0.9644
Predicted R^2^	0.9044
V.%	4.10
Signal-to-Noise Ratio	25.5403

### Appendix A.5. VSM Hysteresis Loop and Zeta Potential Energy

**Table A3:** Zeta potential test results.

Sample	Project	Mean	Standard Deviation	RSD	Minimun	Maximum
BC	Zeta Potential (mV)	−24.29	0.3227	1.329	−24.66	−24.06
	Peak 1 Mean (mV)	−24.29	0.3227	1.329	−24.66	−24.06
	Conductivity (mS/cm)	1.41	2.719 × 10^−16^	1.928 × 10^−14^	1.41	1.41
	Wall Zeta Potential (mV)	−15.76	0.2968	1.883	−16.1	−15.56
	Zeta Deviation (mV)	4.055	03029	7.469	3.745	4.351
	Derived Mean Count Rate (kcps)	2756	5.569 × 10^−13^	2.021 × 10^−14^	2756	2756
	Reference Beam Count Rate (kcps)	2047	0	0	2047	2047
	Quality Factor	2.952	0.7044	23.86	2.222	3.627
FCBC	Zeta Potential (mV)	41.64	1.854	4.452	40.1	43.7
	Peak 1 Mean (mV)	13.51	46.95	347.5	−40.7	41.13
	Zeta Peak 2 Mean (mV)	−31.61	-	-	−31.61	−31.61
	Conductivity (mS/cm)	2.715	0	0	2.715	2.715
	Wall Zeta Potential (mV)	24.03	2.945	12.26	21.46	27.24
	Zeta Deviation (mV)	36.28	52.61	145	5.285	97.02
	Derived Mean Count Rate (kcps)	4918	0	0	4918	4918
	Reference Beam Count Rate (kcps)	2136	0	0	2136	2136
	Quality Factor	4.193	1.814	43.28	2.104	5.38
